# Asynchronous Replication and Autosome-Pair Non-Equivalence in Human Embryonic Stem Cells

**DOI:** 10.1371/journal.pone.0004970

**Published:** 2009-03-27

**Authors:** Devkanya Dutta, Alexander W. Ensminger, Jacob P. Zucker, Andrew Chess

**Affiliations:** Center For Human Genetic Research and Department of Medicine, Massachusetts General Hospital, Harvard Medical School, Simches Research Center, Boston, Massachusetts, United States of America; Centre National de la Recherche Scientifique, France

## Abstract

A number of mammalian genes exhibit the unusual properties of random monoallelic expression and random asynchronous replication. Such exceptional genes include genes subject to X inactivation and autosomal genes including odorant receptors, immunoglobulins, interleukins, pheromone receptors, and p120 catenin. In differentiated cells, random asynchronous replication of interspersed autosomal genes is coordinated at the whole chromosome level, indicative of chromosome-pair non-equivalence. Here we have investigated the replication pattern of the random asynchronously replicating genes in undifferentiated human embryonic stem cells, using fluorescence *in situ* hybridization based assay. We show that allele-specific replication of X-linked genes and random monoallelic autosomal genes occur in human embryonic stem cells. The direction of replication is coordinated at the whole chromosome level and can cross the centromere, indicating the existence of autosome-pair non-equivalence in human embryonic stem cells. These results suggest that epigenetic mechanism(s) that randomly distinguish between two parental alleles are emerging in the cells of the inner cell mass, the source of human embryonic stem cells.

## Introduction

A number of genes in the mammalian genome exhibit monoallelic transcription [1]. These genes fall into three distinct classes. One class is the autosomal imprinted genes, such as *IGF2* and *H19*, which are monoallelically expressed in a parent-of-origin-specific manner [2]. By contrast, the second and third class are comprised of genes that are randomly monoallelic. For these genes, individual cells randomly choose one of the two alleles for transcription, and the choice is epigenetically maintained in each cell's progeny [3], [4]. The second class is comprised of genes subject to X chromosome inactivation. X inactivation in female cells is a random process resulting in half of the cells inactivating the maternal X chromosome and half inactivating the paternal X chromosome [5]. The third class consists of the randomly monoallelic autosomal genes. Typical examples of members of this group includes genes coding for the large family of odorant receptor genes [6], the immunoglobulins [7], T cell receptors [8], interleukins [9]–[11], natural killer cell receptors [12] and pheromone receptors [13], [14]. A recent study has expanded the repertoire of genes of this class; using a genome-wide approach, we have identified more than 300 autosomal genes displaying random monoallelic expression, suggesting that this unusual mode of gene transcription is more widespread in autosomes than previously thought [15].

As far as it has been studied, all monoallelically expressed genes exhibit the unusual property of asynchronous replication [6], [10], [16], defined as one allele replicating during an earlier portion of S-phase than the other allele. Asynchronous replication appears to be set up early in development [16], [17], and is found even in tissues in which the genes are not expressed [6], [18]. For example, asynchronous replication of odorant receptor genes has been observed in all cell types analyzed including lymphoblasts and fibroblasts [3], [6]. For the randomly monoallelic genes, i.e. the X-linked genes and the autosomal random monoallelic genes, the choice of the early replicating allele is also random. On both the X chromosome and the autosomes, this choice follows the so called “n-1” rule where all but one allele replicates late [18], [19]. Notably, once the random decision of the early replicating allele is made within a cell, it is heritable in its progeny [3], [4]. A striking feature of random asynchronously replicating genes is chromosome-wide coordination. Each cell randomly chooses either the maternal or paternal copy of each autosome pair, such that alleles of these genes scattered across the chosen chromosome are earlier replicating than the alleles on the homologous chromosome. This observation, made both in mouse and human cells [3], [18], indicates a chromosome-pair non-equivalence for autosomes, similar to X-chromosome inactivation observed in female cells.

The mechanisms that establish allelic control of replication timing are poorly understood. Developmental studies in mice show that asynchronous replication of autosomal genes occurs in the zygotic stage post-fertilization, but is subsequently erased in the morula and blastula stages of the embryo [16], [17]. Asynchronous replication is observed in mouse embryonic stem cells [3], [16], which mimic the post-blastula stage embryo. These results suggest that random asynchronous replication is set in the developing mouse around the time of implantation.

In human, we have shown the occurrence of allele-specific replication and autosome-pair non-equivalence in adult somatic cells [18]. Here we asked whether these events are also observed in the human embryonic stem (ES) cells. Mouse and human ES cells have been shown to behave differently in many aspects of gene regulation, including the epigenetic mechanism of X inactivation [20]–[25]. Our study aimed at investigating whether such differences extend to the replication pattern of randomly monoallelic genes.

Our results show that allele-specific replication of X-linked genes and random monoallelic autosomal genes is established in the undifferentiated human ES cells H7 and H9. Using the newly identified monoallelic gene *amyloid precursor protein* (*APP)*, we demonstrate that asynchronous replication is random with respect to parent-of-origin in human ES cells. We also show that the direction of replication is coordinated at the whole chromosome level and can cross the centromere. Taken together, these data indicate that autosome-pair non-equivalence exists in human ES cells.

## Materials and Methods

### Cell culture

H9 and H7 cells (obtained from WiCell) were cultured on irradiated mouse embryonic fibroblast (MEF) feeder layer (obtained from day 13.5 embryos). The cells were grown in 80% KnockOut™ DMEM medium (Invitrogen), supplemented with 10% KnockOut™ SR- a serum replacement formulation (Invitrogen), 10% Plasmanate, a human plasma protein fraction (Bayer Corp.), 1% non-essential amino acid (Invitrogen), 2mM glutamine, 0.05 mM 2-mercaptoethanol (Invitrogen), penicillin (100U/ml), Streptomycin (100 μg/ml) (Invitrogen) and 10 ng/ml of basic fibroblast growth factor (bFGF) (Invitrogen). Cultures were passaged by 0.05% Trypsin/EDTA at a 1:3 or 1:4 ratio upon confluence. Both cell lines were differentiated by EB suspension in low-attachment six-well plates (Corning) in media containing 20% FBS (Hyclone) in place of the serum replacement, plasmanate and FGF. The primary human fibroblast cell line, WI-38 (American Type Culture Collection) was maintained under standard conditions (88% DMEM, 1% Penicillin-Streptomycin, 10% FBS). For FISH assay, cells were treated as described previously [3]. Briefly, cells were pulse-labeled with BrdU for 45-50 minutes, followed by fixation in 3:1 methanol-acetic acid. Fixing cells using methanol disrupts their nuclear architecture, and minimizes the contribution of sister chromatid cohesion. A more detailed discussion of this assay and its comparison with other FISH protocols, used for studying sister chromatid cohesion, has been described in a previous paper published by our group [18].

### Cytogenetic Analysis

Karyotype analysis on H7 and H9 cell lines were carried out via g-banding and was conducted, at or close to the initiation of FISH experiments, by the Cytogenetics Laboratory, Tufts Medical Center, Boston, USA. For H9 line, 15 cells were assessed and for H7 line, 20 cells were assessed. For H9 cell line, all the cells analyzed showed an abnormal karyotype (Figure S1 in supplementary information).

### FISH

FISH analysis was performed as previously described [18]. Briefly, large PCR products were used as probes against the different loci. BACs were obtained from Invitrogen and served as templates for the PCR reactions. The BACs used for the different probes are as follows: RP11-1105A14 (*OR10A3*), RP11-1114G15 (*OR7D2*), RP11-1109J16 (*OR10B1P*), RP11-158C6 (*OR2AT4*), RP11-381F14 (*OR5AH1P*), RP11-111N23 (*OR4X2*), RP11-261F13 (*IL1F9*), RP11-17K19 (*IL5*), RP11-117N12 (*IL12B*), RP11-35O20 (*IL16*), RP11-344F17 (constant region of *IGK*), RP11-410J1 (*APP*), RP11-318G17 (*PPEF1*), RP11-42E12 (*DMD*), RP11-91G17 (*LARP*), RP11-10I9 (*C9ORF43*), RP11-299H21 (*C40*). Primers were designed to produce a 9000-11000 bp product using the Advantage 2 PCR system (BD Biosciences Clontech). The sequences of the primers are provided as supplementary information in Table S1. PCR product for each gene, except for *IL5*, was amplified from the coding region. For *IL5*, PCR product from 9 Kb upstream of the interleukin's coding region was used. PCR products were purified using the Wizard PCR preps DNA Purification System (Promega). For fluorescent probe preparations, 1 mg of DNA was directly labeled with either Cys-dCTP or with FluorX-dCTP using a Nick Translation kit (Amersham Biosciences). Labeled probes were purified using G-50 Sephadex columns (Roche), precipitated with 30 mg human cot-1 DNA and 70 mg Salmon sperm DNA (Invitrogen), washed in 75% ethanol followed by 100% ethanol and resuspended in 100μl hybridization buffer (50% formamide, 10% dextran sulfate, 1X SSC). 10μl aliquote of each probe was pre-hybridized (90°C for 5 min, followed by 10min at 37°C) and then hybridized overnight with cells dropped on poly-L-Lysine coated slides. Subsequent washes and antibody detection of BrdU were as previously described [3]. The cells were mounted in Vectashield mounting medium containing DAPI (Vector Laboratories) to counter-stain the nuclei. For replication timing analysis, only BrdU positive cells were counted. Cells were viewed with a Nikon E600 fluorescent microscope. Images were captured with a CCD camera using SPOT Advanced software.

### Immunostaining

Undifferentiated ES cells were fixed and immunostained by standard immunocytochemistry. Briefly, cells were rinsed with 1× PBS, fixed in 4% paraformaldehyde for 25–30 minutes at room temperature, washed three times with 1× PBS (5 minutes each wash), and then blocked overnight at 4°C with blocking solution (1× PBS, 0.1% Triton-X 100, 5% normal donkey serum). Next, cells were incubated with the primary antibody at appropriate dilution and incubated overnight at 4°C with gentle rocking. The primary antibodies used were : mouse monoclonal OCT-4 (Santa Cruz) at 1∶200, rat monoclonal SSEA-3 (Developmental Hybridoma Studies Bank) at 1∶500, mouse monoclonal SSEA-4 (Developmental Hybridoma Studies Bank) at 1∶500, mouse monoclonal TRA-1-60 (Chemicon International) at 1∶500, mouse monoclonal TRA-1-81 (Chemicon International) at 1∶500. Next, cells were washed with the blocking solution and incubated for two hours with appropriate fluorescent secondary antibodies: Rhodamine-Red-X-donkey anti-mouse IgG (Jackson ImmunoResearch) at 1∶200 or Rhodamine-Red-X-goat-anti-rat (Jackson ImmunoResearch) at 1∶200. The cells were washed three times with PBS, with the second wash containing DAPI at a final concentration of 1μg/ml.

### *P*-values

*P*-values were calculated based on a binomial probability distribution.

## Results

### Asynchronous replication of random monoallelic autosomal genes and X-linked genes in the human ES cells

We examined the replication timing of X-linked genes and autosomal random monoallelic genes in the human ES cells by using fluorescence *in situ* hybridization (FISH) based assay [26]. In this assay, the numbers of hybridization signals for a locus of interest are counted in S-phase interphase nuclei labeled with BrdU. Some cells in the population will display two single hybridization dots, indicating that neither allele has replicated (a single-single or SS pattern), while cells of a second class will display two double dots, indicating that both alleles have replicated and have sufficiently separated (a double-double or DD pattern). A third class of cells will have one single dot and one double dot, indicating replication of only one of the two alleles (a single-double or SD pattern). For most genes, whose alleles are synchronously replicated, the percentage of S-phase cells showing an SD pattern is relatively low (about 15–20%). At the same time, asynchronously replicating genes reveal a higher proportion of cells with an SD pattern (35–50%). Therefore, for a particular locus, counting the percentage of S-phase cells with an SD pattern tells us whether it is synchronously replicating or asynchronously replicating in the population of cells. Note that, this FISH-based assay of replication timing involves stringent cell fixation and denaturation conditions that disrupt nuclear structures, thereby minimizing the contribution of sister chromatid cohesion. Though this assay does not directly measure replication timing (for instance by assessing BrdU incorporation), it is an accurate indicator of asynchronous replication; it has been corroborated by direct measurements of DNA replication by our lab and others [3], [16], [27].

**Figure 1:**
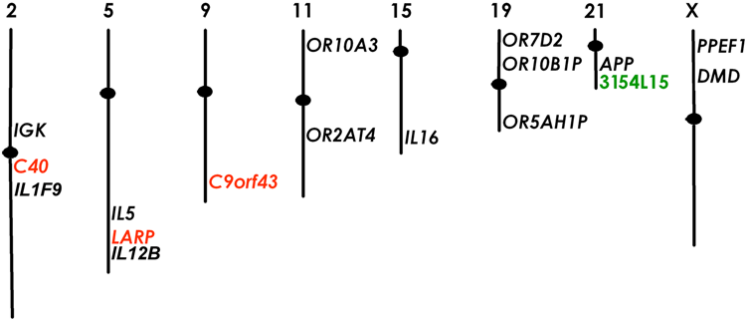
Chromosomal location of the probes analyzed in this study. The asynchronously replicating probes are represented in black and the synchronously replicating control probes are represented in red. The BAC CTD-3154L15, used for studying the random replication of *APP*, is shown in green.

Using this assay we studied the replication pattern of a number of monoallelic loci in the female human ES cell lines H9 and H7. We looked at six odorant receptor genes (*OR10A3, OR7D2, OR10B1P, OR2AT4, OR5AHIP, OR4X2*), four interleukin genes (*IL1F9, IL5, IL12B, IL16*), the κ-immunoglubulin gene (*IGK*) and two X-linked genes (*PPEF1, DMD*). These genes have been shown to replicate asynchronously in mature human cells [18]. In addition, we also studied the replication pattern for the gene coding for the amyloid precursor protein (*APP*), which we have recently shown to be monoallelically expressed [15]. Since all monoallelically expressed genes examined so far have the property of asynchronous replication, we tested *APP* for asynchronous replication. For control studies, primary human fibroblast cell line WI-38 was used as a control cell line and probes against three known synchronously replicating loci *LARP, C9ORF43* and *C40* [18] were used for testing synchronous replication in the human ES cells. The relative locations of the loci on different chromosomes are represented schematically in Figure 1.

**Figure 2:**
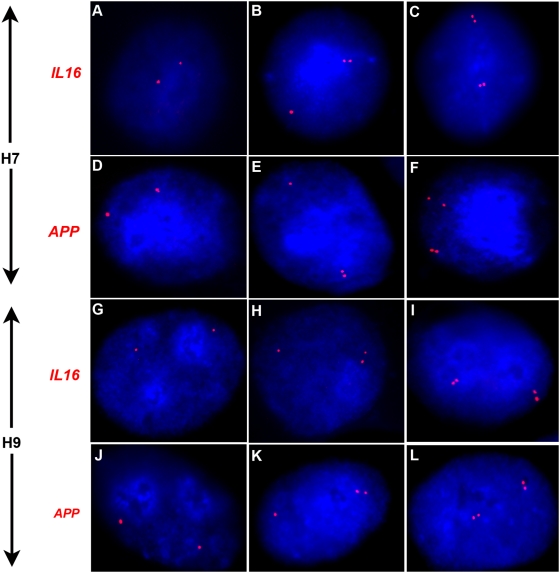
FISH images confirming asynchronous replication in human ES cells. Nuclei are stained with DAPI (blue) and hybridized with Cy3-labeled florescent probe (red). (A–F) FISH images of nuclei from H7 cells hybridized with probes against *IL16* (A–C) and *APP* (D–F). Panels A, B, C show SS, SD and DD patterns, respectively, in nuclei of cells hybridized with a probe against *IL16*. Images in panels D, E and F show SS, SD and DD patterns, respectively, of nuclei hybridized with a probe against *APP*. (G–L) Nuclei of H9 cells hybridized with probes against *IL16* (G–I) and *APP* (J–L). Images G, H, I show SS, SD and DD patterns of hybridization, respectively, for the probe *IL16* while J, K and L show SS, SD and DD pattern of hybridization for *APP* in nuclei of H9 cells.

FISH assays done with probes against the monoallelic genes showed a high percentage of S-phase cells (∼40–50%) having the SD pattern in both H7 and H9 lines (Table 1), indicating that these genes replicate asynchronously in human ES cells. It is interesting to note that *APP*, identified as monoallelic from the recent genome-wide survey [15], confirmed our expectation as an asynchronously replicating gene in the differentiated human cell line WI-38 and also in human ES cells. FISH images for two of the probes, *IL6* and *APP* are shown in Figure 2.

**Table 1:** Percentage SD counts in WI-38, H7 and H9 cells.

	WI-38	H7	H9
***OR10A3***	53	46	49
***OR7D2***	46	42	46
***OR10B1P***	44	49	50
***OR2AT4***	44	41	47
***OR5AH1P***	47	39	43
***OR4X2***	44	44	30
***IL1F9***	43	50	40
***IL5***	42	38	46
***IL12B***	45	46	46
***IL16***	40	40	39
***IGK***	42	50	43
***APP***	48	45	51
***PPEF1***	49	44	50
***DMD***	44	48	47
***LARP***	22	24	18
***C9orf43***	25	25	23
***C40***	19	21	24

The asynchronously replicating probes show SD counts between 38–51% in H7 and H9 cells, showing that they are also asynchronous in the embryonic stem cells. The synchronously replicating loci, *LARP*, *C40* and *C9orf43* have lower SD counts in the ES cells. For each probe 100 cells were counted per cell line.

**Figure 3:**
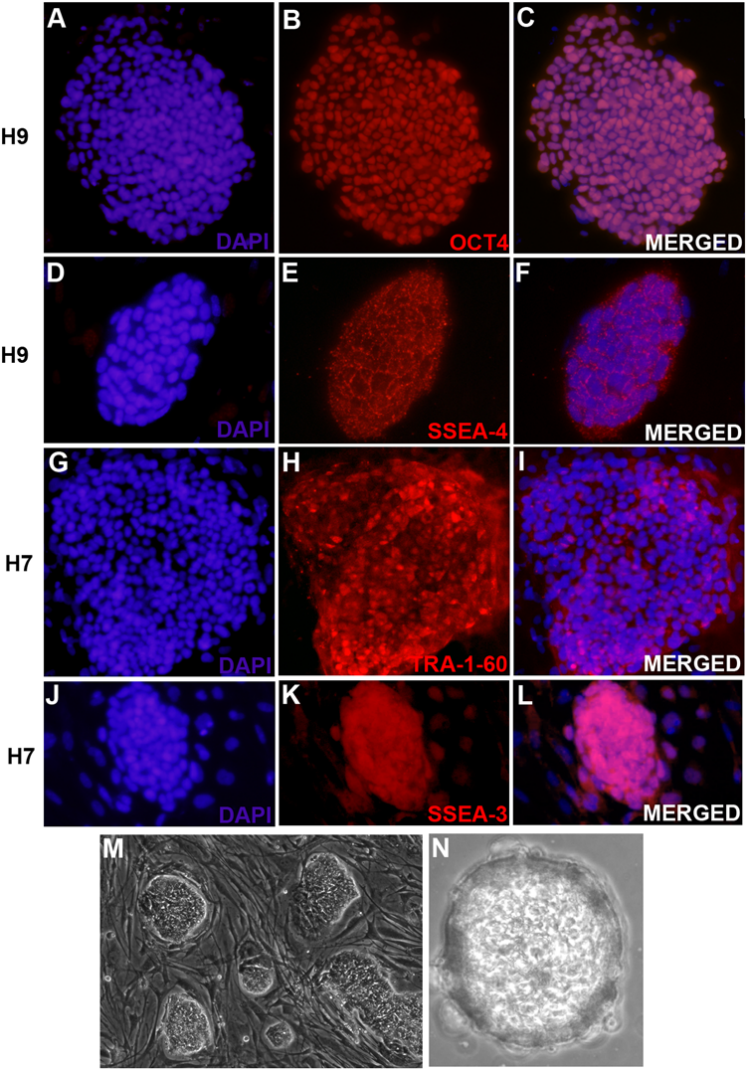
Characterization of undifferentiated state of H7 and H9 cells. (A–F) Immunostaining of undifferentiated H9 cells with antibodies against OCT4 (A–C) and SSEA-4 (D–F). (A, B, C) Nuclei in a colony of H9 cells stained simultaneously with DAPI (A) and an antibody against OCT4 (B). C is the merged image of A and B showing all nuclei stained with OCT4 antibody. (D, E, F) Nuclei of a colony of H9 cells stained with DAPI (D) and anti-SSEA-4 antibody (E). F is the merged image of D and E. (G–L) Immunostaining of undifferentiated H7 cells with antibodies against TRA-1-60 (G–I) and SSEA-3 (J–L). (G, H, I) H7 nuclei stained with DAPI (G) and anti-TRA-1-60 antibody (H). I is the merged image of G and H. (J,K,L) Nuclei of a colony of undifferentiated H7 cells labeled with DAPI (J) and anti-SSEA-3 antibody (K). L is the merged image of J and K. (M, N) Embryoid body formation in H9 cells. (M) Colonies of undifferentiated H9 cells, observed under light microscope. (N) Picture of an embryoid body (on day 21) formed upon growing the cells in differentiation medium in low attachment plates.

The H7 and H9 cell lines used in our study were karyotyped by standard G-banding. The H7 line showed a normal karyotype. The H9 cells, on the other hand, showed an abnormal karyotype characterized by the presence of an unbalanced translocation involving chromosomes 17 and 21 (supplementary Figure S1). One copy of chromosome 21 has an additional copy of part of the long arm of chromosome 17 replacing the distal region. The net result is trisomy for 17q21 to qter, and monosomy for 21q from 21q22 to qter. Recurrent gain of chromosome 17q in human ES cell lines has been reported earlier [28]. However, despite this abnormality, the results of replication pattern in H9 cells were comparable with that observed in H7 cells. The undifferentiated state of the human ES lines was confirmed by staining with antibodies against pluripotency markers OCT4, TRA-1-60, TRA-1-81, SSEA-3 and SSEA-4 **(** Figures 3A–3L). The ES lines were also tested for ability to form embryoid bodies upon induction of differentiating conditions (Figures 3M, 3N).

**Figure 4:**
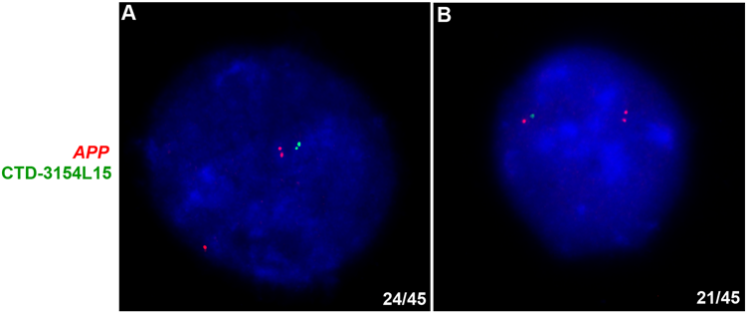
Random asynchronous replication of *APP* in H9 cells. H9 cells were simultaneously hybridized with a probe against *APP* (red) and a FluorX-dCTP labeled BAC CTD-3154L15 (green). The blue color depicts nuclear DAPI staining. The H9 cells have a heterozygous deletion in chromosome 21 (from 21q22 to qter). The BAC CTD-3154L15 maps within this deleted region, and was used to mark one of the two copies of chromosome 21. The *APP* gene on chromosome 21 resides close to, but doesn't fall within, the deleted region. The images in A and B show nuclei from two different cells, from a same population, each of which has replicated a different allele early. (A) Nucleus of an H9 cell in which the double dot signals for *APP* and CTD-3154L15 are in close proximity, indicating that the *APP* allele residing in the intact chromosome has replicated early. (B) Nucleus of an H9 cell in which the double dot signal of *APP* does not have a signal for the BAC in its proximity, showing that the *APP* allele linked to the deleted chromosome has replicated early. In 45 cells counted, 24 cells replicated the intact chromosome early (like the cell in A), whereas 21 had replicated the allele linked to the deletion early (like the cell in B).

Asynchronous replication of random monoallelically expressed autosomal genes is established in a stochastic manner: some cells have an early replicating maternal allele and others have an early replicating paternal allele [3], [16]. This is in contrast to observation of imprinted genes where asynchronous replication is parent-specific [17]. We wanted to examine whether asynchronous replication of random monoallelic genes is random in human ES cells, as had been observed in human fibroblasts [18]. For our analysis we selected the differentially replicating *APP* as the candidate gene and the nonclonal ES cell line H9. As mentioned earlier, the H9 cells have a heterozygous deletion in chromosome 21, from 21q22 to qter. This deletion served as a mark for one of the two copies of chromosome 21. The *APP* gene resides on the same arm of chromosome 21 (21q21.3) but is not within the deleted region. Two-color FISH was performed using a red probe against the *APP* locus and a green labeled BAC probe, CTD-3154L15, which maps within the deleted region (21q22.3). We scored 45 cells which showed a SD pattern for *APP*. Out of these 45 cells, we observed 24 cells who had nuclei in which the early replicating allele of *APP* is linked to the deletion, while the remaining 21 cells had nuclei in which the early allele of *APP* resided on the intact copy of chromosome 21 (Figure 4). This shows that allele-specific replication of the *APP* locus is random with respect to parent-of-origin in undifferentiated human ES cells.

### Coordination of asynchronous replication in human ES cells

The observation of asynchronous replication of autosomal genes in ES cells led us to examine whether the direction of replication was subject to chromosome-wide coordination. The level of coordination of two distant genes on a particular chromosome was examined by using two-color FISH analysis and scoring cells which displayed a single-double (SD) signal for both genes [3], [18]. This type of pattern can be found if the two genes replicate in an overlapping portion of S-phase. If the two genes are coordinated, the double dots for both genes should reside on the same chromosome and thus will be near each other in the nucleus. If the two genes are not coordinated, then the double dots for both genes should be on the same chromosome only 50% of the time. The effectiveness of the two-color FISH assay depends on the physical proximity of two linked loci within the nucleus. If the two probes are more than 50Mb, the feasibility of the assay begins to diminish as signal coming from the paternal allele of one gene may be closest to the maternal allele of the other gene.

**Figure 5:**
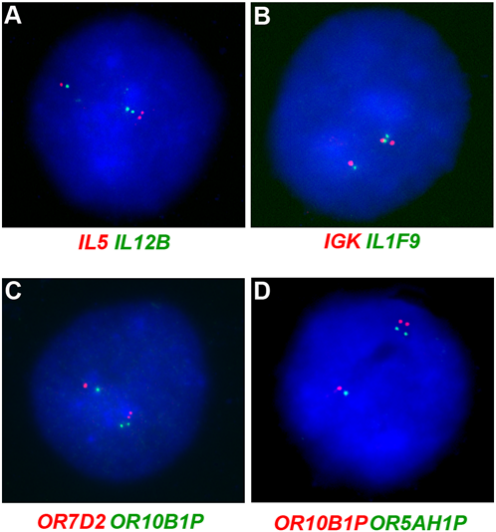
Coordination of asynchronous replication. Two-color FISH analysis was done in H7 cells to show coordination of asynchronous replication in H7 cells. Blue represents DAPI staining of the nucleus. (A) Nucleus of an H7 cell labeled with probes against *IL5* (red) and *IL12B* (green). The double dot signals for both red and green probes are in close proximity, indicating that they are on the same chromosome. 27 out of 33 cells showed such a pattern, indicating coordination in replication of *IL5* and *IL12B.* (B) Nucleus of an H7 cell labeled with probes against *IGK* (red) and *IL1F9* (green). Similar to the cell in A, in this cell also the double dot signals for both the probes reside on the same chromosome. 26 out of 32 cells showed this pattern, indicating coordination of replication of *IGK* and *IL1F9.* (C) Two odorant receptor genes on chromosome 19, *OR7D2* (red) and *OR10B1P* (green) show a similar coordinated pattern of replication (21 out of 25 cells). (D) Likewise, the odorant receptor genes *OR10B1P* (red) and *OR5AH1P* (green), on chromosome 19, are coordinated (31 out of 40 cells).

Using this two-color approach, we sought to determine the level of coordination in H7 cells (Figure 5). We analyzed *IL5* and *IL12B,* two interleukin genes located 26.8Mb apart on chromosome 5. The synchronously replicating gene *LARP* resides between these two genes. Two-color FISH assay in H7 cells showed 27 out of 33 cells having double dot signals for both these genes residing on the same chromosome, thereby revealing coordination (P<0.001) (Figure 5A). We next analyzed two genes which reside on different arms of their resident chromosome. *IGK* and the interleukin gene *IL1F9* are located on opposite sides of the centromere on chromosome 2 and are 22.2 Mb apart. We observed coordination in 26 out of 32 H7 cells (P<0.001) (Figure 5B), demonstrating that coordination extends across the centromere and most likely reflects a chromosome-wide choice. To confirm that coordination is indeed chromosome-wide, we next analyzed three asynchronously replicating odorant receptor genes residing on chromosome 19: *OR7D2*, *OR10B1P* and *OR5AH1P*. We chose these three loci as they extend on both arms of chromosome 19 and together cover 83% of this chromosome. *OR7D2* and *OR10B1P* are located 6 Mb apart while *OR10B1P* and *OR5AH1P* are 46.8 Mb apart. We observed evidence of coordination between *OR7D2* and *OR10B1P* (21 out of 25 H7 cells, P<0.001) (Figure 5C). Similarly, we observed coordination between *OR10B1P* and *OR5AH1P* (31 out of 40 H7 cells, P<0.001) (Figure 5D). This implies that *OR7D2* and *OR5AH1P* are also coordinated with each other, thereby indicating that all three of these loci, covering most of chromosome 19, are coordinated. These results indicate that random asynchronous replication is coordinated across entire chromosomes in human ES cells.

## Discussion

In this study we have shown that for both autosomal monoallelically expressed genes and for X-linked genes, there is random asynchronous replication in human ES cells. We also showed that the asynchronous replication of autosomal genes on a given chromosome is coordinated, indicating that the autosomal pair non-equivalence is already established in the human ES cells.

With the potential to give rise to all types of somatic cells, human ES cells have generated enormous interest as agents of cell replacement therapy. However, it is increasingly becoming clear that these cell lines exhibit substantial fluidity in their properties [29] and need to be studied thoroughly for genetic and epigenetic stability before being utilized in vivo. Studies of different epigenetic events in human ES cells have revealed different trends. Examination of the expression of imprinted genes have shown that nearly all human ES cell lines possess a substantial degree of stability in terms of genomic imprinting status [30]. On the other hand, a study of genome-wide DNA methylation profile identified substantial DNA methylation instability within different human ES cell epigenome [31]. Similarly, epigenetic anomalies have been identified in X chromosome inactivation studies in human ES cells. Results from three separate groups have shown that different female ES cell lines exhibit variation in X chromosome inactivation markers like *XIST* RNA expression and H3K27 trimethylation status [23]–[25]. Interestingly, regardless of the presence or absence of X chromosome inactivation markers, all female ES cells exhibited a monoallelic expression pattern for a majority of X-linked genes, indicating that they have already established X chromosome inactivation during the process of derivation and/or propagation. In light of these recent studies, our study of asynchronous replication in human ES cells adds significant weight towards further obtaining a more complete picture of the biology of ES cells.

The mechanisms that establish and maintain asynchronous replication between parental alleles are not yet clearly delineated. Given the diverse repertoire of genes that manifest asynchronous replication, it is likely that different mechanisms are at play. Differences in replication timing could be a consequence of local or broad differences in chromatin structure. Another possibility is the correlation between subnuclear localization and replication timing. A study by Gribnau et al indicated that distinct nuclear compartments with different replication characteristics regulate asynchronous replication timing for the imprinted locus *Igf2-H19* locus in mouse [27]. The two *Igf2-H19* loci are localized in different subnuclear compartments, having distinct replication timing characteristics, and therefore replicated asynchronously in S-phase**.** To further understand the mechanisms involved in allele-specific replication, it is important to identify the molecular regulators of asynchronous replication. Again, the *Igf2-H19* domain is the most studied locus in this line of investigation. Asynchronous replication of the *Igf2-H19* locus is not affected by absence of DNA methylases *Dnmt1* or *DNmt3L*, indicating differential DNA methylation is not involved in asynchronous replication of this locus [27]. A recent study has identified the insulator protein CTCF to be a regulator for asynchronous replication of the *Igf2-H19* domain [32]. Whether these results holds true for the autosomal random asynchronously replicating genes remains to be investigated. It is noteworthy that in many autosomal chromosomes (for e.g., chromosome 11 in human, chromosome 7 in mouse), random asynchronously replicating loci are interspersed with imprinted gene regions. For imprinted genes, direction of asynchronous replication is not random and is determined by parent-of-origin specific manner [17]. Therefore, these imprinted regions are regulated independently of the mechanisms that establish asynchronous replication of the rest of the random asynchronously replicating genes.

For the autosomal random asynchronous genes, superimposed upon the mechanism of asynchronous replication must be a mechanism that regulates chromosome-wide coordination. How can large interspersed replication domains on the same chromosome coordinate whether to replicate early or replicate late? One possible model for autosomal coordination is that, similar to *XIST* in X inactivation, non-coding transcripts propagate random choice made at a discrete part of an autosome, to the far reaches of that homolog. This model prompted us to perform a microarray screen to search for nuclear-enriched non-coding RNAs, using *XIST'*s properties as a guide. The screen identified two unique non-coding polyadenylated RNAs which, like *XIST*, were significantly enriched in the nucleus: *Nuclear Enriched Abundant Transcripts 1* and 2 (*NEAT1* and *NEAT2*) [33]. The broad distribution of these two RNAs, however, indicated that they are not involved in regulation of replication of its parent autosome. These RNAs were found to be associated with ubiquitous SC35 splicing domains, and their functions are linked to the mechanism of pre-mRNA metabolism [33]. In spite of these observations, the role of a yet-to-be-identified non-coding RNA(s) in regulating autosomal coordination can not be ruled out. A second model for coordination is the involvement of a physical interaction between “coordination centers” on the homologous chromosomes. Such a mechanism of homologous chromosomal pairing has been recently identified in X inactivation [34], [35]. Transient, physical “pairing” of the *Xic* region of the two X chromosomes before the onset of X inactivation has been proposed to regulate counting and mutually exclusive choice during X inactivation. Further studies are needed to elucidate the mechanisms underlying chromosome-wide coordinated asynchronous replication. Our study is a first step towards using ES cells as model system to explore the significance of asynchronous replication in these cells and differentiated cells.

Understanding the mechanism of asynchronous replication gains added importance with the recent identification, by our group, of multiple autosomal genes to be monoallelically expressed [15]. Many of these genes, *APP* gene being one of them, are involved in human diseases. Since all monoallelically expressed genes tested so far also replicate asynchronously, we assume that all these newly identified autosomal genes are also asynchronously replicating. In the present study we have confirmed the allele-specific replication of *APP* gene in undifferentiated human cells. Using the *APP* gene, it would be interesting to further explore the possible link between asynchronous replication and pathological conditions in human.

## Supporting Information

Figure S1 Karyotype analysis by G-banding was performed on 15 metaphase spreads of cells ( passage number = 39). All 15 spreads showed the presence of an unbalanced translocation involving chromosomes 17 and 21. One copy of chromosome 21 (indicated by black arrow) has an additional copy of part of the long arm of chromosome 17 replacing the distal region. The net result is trisomy for 17q21 to qter, and monosomy for 21q from 21q22 to qter
(0.19 MB TIF)

Table S1 Sequences of the forward and reverse primers used for PCR amplification to make FISH probes against the corresponding genes
(0.04 MB DOC)
