# Supplementary figures and images for: Asynchronous Replication and Autosome-Pair Non-Equivalence in Human Embryonic Stem Cells
Source: PLoS One. 2009 Mar 27;4(3):e4970. doi: 10.1371/journal.pone.0004970 (PMC2657208; doi:10.1371/journal.pone.0004970)

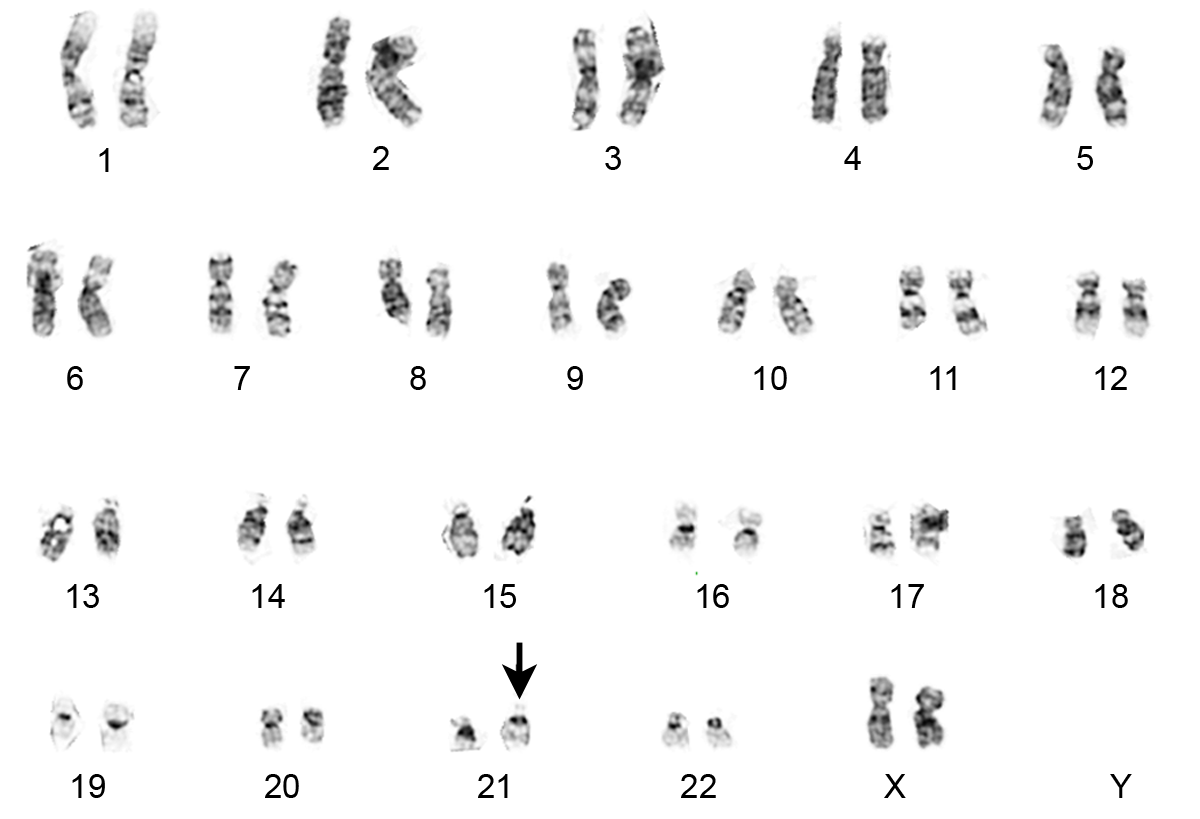

Supplement: Figure S1 — Karyotype analysis by G-banding was performed on 15 metaphase spreads of cells ( passage number = 39). All 15 spreads showed the presence of an unbalanced translocation involving chromosomes 17 and 21. One copy of chromosome 21 (indicated by black arrow) has an additional copy of part of the long arm of chromosome 17 replacing the distal region. The net result is trisomy for 17q21 to qter, and monosomy for 21q from 21q22 to qter (0.19 MB TIF) [file pone.0004970.s001.tif]
