# Supplementary material for: Asynchronous Replication and Autosome-Pair Non-Equivalence in Human Embryonic Stem Cells
Source: PLoS One. 2009 Mar 27;4(3):e4970. doi: 10.1371/journal.pone.0004970 (PMC2657208; doi:10.1371/journal.pone.0004970)
Supplement: Table S1 — Sequences of the forward and reverse primers used for PCR amplification to make FISH probes against the corresponding genes (0.04 MB DOC) [file pone.0004970.s002.doc]

| **Gene** | **Forward Primer** | **Reverse Primer** |
| --- | --- | --- |
| *OR10A3* | CATCGCTCCCAGGAGAAAACATTCAGT | GAGGTGCTTCTGGGGTTTCAGTGGTTA |
| *OR7D2* | gaagaatttggcaggagacgactggac | gcattcaggaagccctcaggaaaAGTT |
| *OR10B1P* | GGAAGGGCCTATGGCCCCTAATAAAGA | ATCTCAACCCTTGGCTTCCTCTGTCAA |
| *OR2AT4* | ATAAGGACAGCATCTTGGGGACAGTGG | ACATCCTGCTGATTGGTAGAGCCCAGT |
| *OR5AH1P* | TGAGGCCAGGTTCCTTCTATCTCGTTG | AGCATAGGGAGGGCCAGAGGTATGAAA |
| *OR4X2* | ACCTGAGATTGTCCCAATCATGGAAGC | CCCTAAAGACTCCACCAAAAGGCTGCT |
| *IL1F9* | AGGAAAATCAGGGATGAAGATGCTGGA | GGGCAGTATCCTCAAGTGGATCTGGAA |
| *IL5* | GGTCTCCAATCCACCCCACCACTAC | TCTGGGAGTACAAAGCAGGAGCATCTAAC |
| *IL12B* | CATTCCATACATCCTGGCAGACAAACG | CCACCCCTTCTCCATGAGAATTTGAGA |
| *IL16* | CCACGTCAGCACCAAGGAGAAAAGAAT | CTCAGGCAGTGTACTAGGCAGCCACAC |
| *IGK* | TAATACCTGGAACAAGGCACGGTGTGA | CTGCTCGAAAAGGGAGTTGAGCTTCAG |
| *APP* | GTGAGGGAGAGGAAGGCATCATGGATA | AAACAGTGAAGGGGTTTCCCAGGTCTC |
| *PPEF1* | CCCAATTCAGATGCAGCTTACCCTTTG | CGGGGCATTAGTTCCATGCCTCTTTAT |
| *DMD* | CACCTGTGTGGGAGAACGAGTGATGTA | AATACAAACCCTGTGGGGAGGTTGATG |
| *LARP* | TTTCCCATTTGATCCTTTAGGGCAAGG | CACCAGGAACCAACAGTGATCCAAGAA |
| *C9orf43* | AGCAAACACATTCATTGCTGGTGGGTA | CCTACTCGTGTTCCTGCGGACTGATTT |
| *C40* | CCCAGACCCGGATTCTTCTAATTCTGG | ACCCATGCTTTTCTTTGCCGAGATGTA |
